# Preparation of Ultrafine W Powder via H_2_ Reduction of Carbon-Containing WO_3_: Influences of Reduction Temperature and C/WO_3_ Molar Ratio

**DOI:** 10.3390/molecules31040658

**Published:** 2026-02-14

**Authors:** Ao-Qi Zeng, Lu Wang, Zheng-Liang Xue

**Affiliations:** 1 Key Laboratory for Ferrous Metallurgy and Resources Utilization of Ministry of Education, Wuhan University of Science and Technology, Wuhan 430081, China; 2 Hubei Provincial Key Laboratory for New Processes of Ironmaking and Steelmaking, Wuhan University of Science and Technology, Wuhan 430081, China

**Keywords:** ultrafine W powder, H_2_ reduction, WO_3_, carbon, reaction mechanism

## Abstract

Ultrafine W powder is a key material for manufacturing high-performance W-based products. In this study, ultrafine W powder was prepared via the H_2_ reduction of carbon-containing WO_3_, and the parameters of reduction temperature (740–830 °C) and C/WO_3_ molar ratio (0.5–2.5) were mainly considered. The results demonstrated that, with the increase in reduction temperature, the reaction rate gradually increased, while the particle size of W powder exhibited a trend showing an initial decrease and then increase, with a minimum value of 146 nm at 770 °C. The results also showed that, with the increase in C/WO_3_ molar ratio, the reaction rate gradually decreased, while the particle size of W powder also first decreased and then increased, with its minimum value at a C/WO_3_ molar ratio of 1.0. The reduction pathways of H_2_ reduction of WO_3_ to W was given as WO_3_→WO_2.9_→WO_2.72_→WO_2_→W. Due to the co-actions of nucleating agent and the synergistic reduction effect, the particle size of W powder obtained by reducing carbon-containing WO_3_ was smaller than that obtained by reducing pure WO_3_, and a possible reaction mechanism was proposed.

## 1. Introduction

Ultrafine W powder possesses high hardness, excellent thermal/electrical conductivity, low sintering temperature, and enhanced thermochemical stability; these attributions allow it to be extensively applied in the fields of aerospace, medical devices, and military industries, etc. [1,2,3,4,5,6]. Therefore, the scale preparation of ultrafine W powder has received increasing attention in recent years.

Numerous preparation methods of ultrafine W powder have been reported [7], such as the high-energy ball milling method [8], the high-temperature self-propagating synthesis method [9], the H_2_ reduction method [10,11], and the cyclic oxidation–reduction method [12]. Among these methods, the H_2_ reduction method dominated [13]. For example, Wang et al. [14] adopted the H_2_ reduction of blue tungsten (WO_2.9_) method to prepare ultrafine W powder, and found that the particle size of W powder increased alongside the increase in H_2_ dew point and sample thickness. Estupinan-Donoso et al. [15] adopted the H_2_ reduction of WO_3_ method and ultrafine W powder with the particle size of 350–600 nm was prepared; also, the results found that water vapor concentration was the main factor affecting the coarsening of the prepared W powder. He et al. [16] studied the effect of cerium doping on the particle size of W powder. The results showed that cerium can act as a nucleation core to boost nucleation, and it can also be uniformly distributed on the particle surface to inhibit its grain growth. Both of the above two mechanisms work together and ultrafine W powder with the particle size of 40–200 nm was prepared.

During the H_2_ reduction process, many studies [17,18] reported that the newly generated water vapor would react with the unreduced W oxides to produce W-O-H volatile compounds, which could cause the growth and coarsening of W powder through the chemical vapor transport (CVT) mechanism. To avoid the occurrence of CVT mechanism, it was found [19] that adding a certain amount of carbon into WO_3_ raw material (defined as carbon-containing WO_3_), may become a potential route for the refinement of W powder. However, the related reduction behavior during the H_2_ reduction of carbon-containing WO_3_ was still insufficient. In order to make up for the gap, the H_2_ reduction of carbon-containing WO_3_ was further investigated, and the influences of reduction temperature and C/WO_3_ molar ratio on the reduction behavior and product characteristics were mainly illustrated.

## 2. Results

### 2.1. TG Analysis

During the H_2_ reduction process, the sample mass was gradually decreased with increased reaction time. Herein, the mass loss (*W_t_*) of sample was calculated by Equation (1), where *m*_0_ and *m_t_* represent the initial mass of sample that reacts for a period of time *t*, respectively.

(1) $$W_{t}=-\frac{m_{t}-m_{0}}{m_{0}}\times 100\%$$

**Figure 1:**
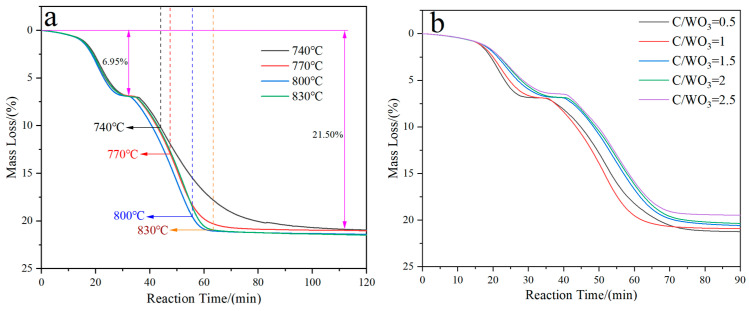
Kinetics curves for the H_2_ reduction of carbon-containing WO_3_: (**a**) Influence of reduction temperature; (**b**) Influence of C/WO_3_ molar ratio.

Figure 1 demonstrates the kinetics curves for the H_2_ reduction of carbon-containing WO_3_ under different conditions. It shows that two plateaus exist for all the four curves; one is located at 6.95 mass%, and the other is located at 21.50 mass%. From Figure 1a, it can also be observed that the lower the holding temperature is, the longer the time required for complete reduction to take place. However, their final mass losses are almost the same and align with the theoretical value (21.70 mass%) for the complete reduction of WO_3_ to W. From Figure 1b it can be seen that the reaction rate for the first plateau decreases with the increase in the C/WO_3_ molar ratio, while no obvious trend is observed for the second plateau.

### 2.2. XRD Analysis

**Figure 2:**
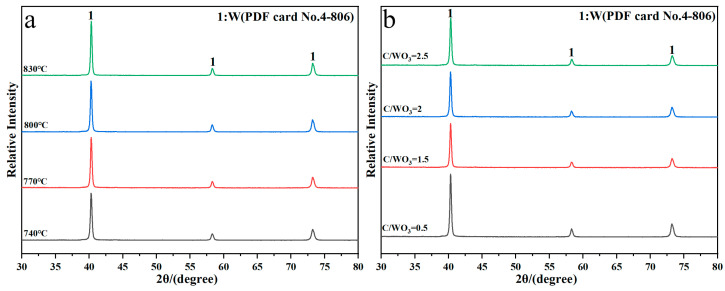
XRD patterns of reaction products obtained by the H_2_ reduction of carbon-containing WO_3_: (**a**) Influence of reduction temperature; (**b**) influence of C/WO_3_ molar ratio.

Figure 2 shows the XRD patterns of reaction products obtained under different conditions. It shows that the diffraction peaks of the reaction products are all indexed to the standard PDF card No. 4-806 of metallic W, without other impurities peaks observed, which indicates that single-phase metallic W is prepared under different reduction temperatures and C/WO_3_ molar ratios. Herein, the crystalline size of the prepared W powders were calculated by the Scherrer Equation (2) [20] and the results found that when the reduction temperatures were 740, 770, 800, and 830 °C, the crystalline sizes were 25.34, 24.40, 24.44, and 25.34 nm, respectively, and when the C/WO_3_ molar ratios were 0.5, 1.5, 2.0, and 2.5, the crystalline sizes were 23.94, 24.23, 23.83, and 22.98 nm, respectively. Because the changes in these crystalline sizes were insignificant, we assumed that no obvious laws existed and the calculated crystalline size of W powder prepared under different conditions can be considered as a constant.

(2) $$D=\frac{K\lambda }{\beta \cos\theta }$$

where *D* is the crystalline size; *K* is the Scherrer constant; *λ* is the wavelength of X-ray radiation; *β* is the full width at the half maximum; *θ* is the Bragg angle.

### 2.3. FE-SEM Observations

**Figure 3:**
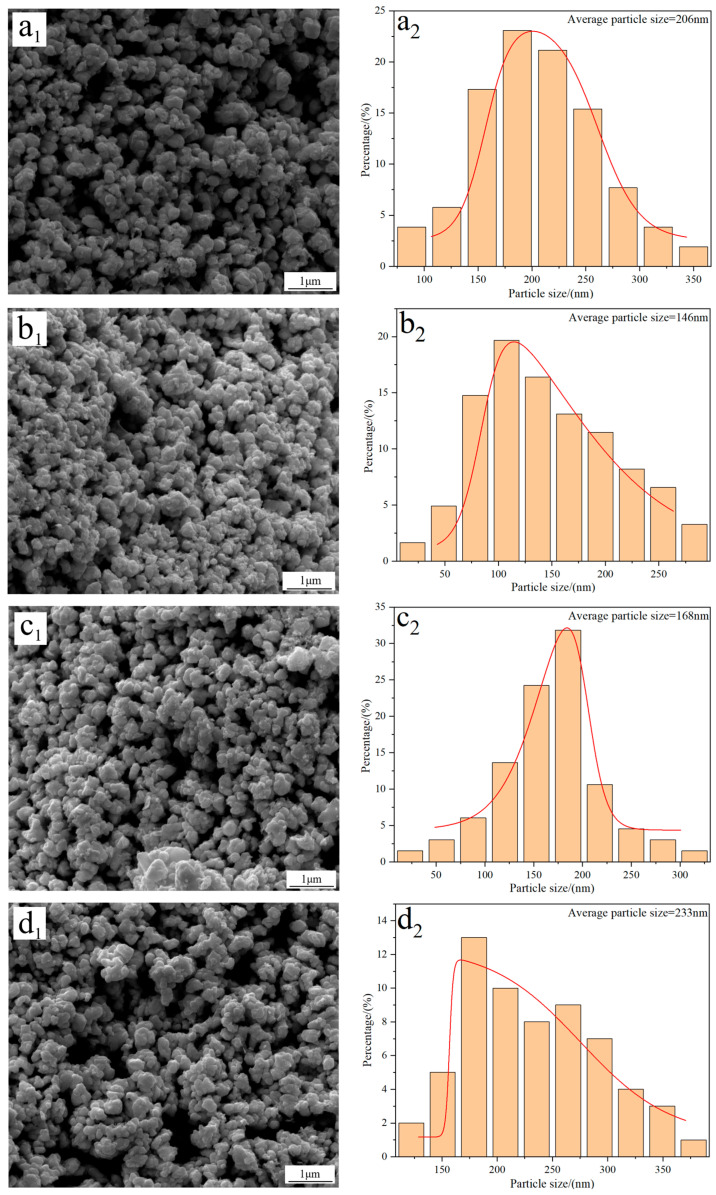
FE-SEM morphology and average particle size of W powders prepared by the H_2_ reduction of carbon-containing WO_3_ at different temperatures: (**a_1_**,**a_2_**) 740 °C; (**b_1_**,**b_2_**) 770 °C; (**c_1_**,**c_2_**) 800 °C; (**d_1_**,**d_2_**) 830 °C. (C/WO_3_ molar ratio: 1.0).

Figure 3 shows the FE-SEM morphology and average particle size of W powders prepared under different reduction temperatures. The results show that there are no significant morphology differences in the W powders, and they all exhibit a spherical/elliptical morphology and a certain amount of agglomeration phenomena. Particle size distribution results reveal that the average particle size of W powder prepared at 740 °C is approximately 206 nm (Figure 3(a_2_)). As the temperature increases to 770 °C, the particle size reduces to 146 nm (Figure 3(b_2_)); however, when the reduction temperature increases to 800 and 830 °C, the particle sizes increase again to values of 168 and 233 nm, respectively (Figure 3(c_2_,d_2_)). The above results demonstrate that the average particle size of W powder shows a trend of initial decrease and then increase with the increase in reduction temperature. Since the minimum particle size of 146 nm was obtained at 770 °C, this temperature was thereby considered as the optimal reduction temperature for preparing ultrafine W powder. One thing that must be noted is that the average particle size of W powder measured by the FE-SEM morphology is larger than that calculated by the XRD pattern; the reason for this difference was due to the serious agglomeration phenomena of the prepared W powder.

With the assumption of the spherical-shaped morphology, the specific surface areas of the W powders prepared at 740, 770, 800, and 830 °C were calculated by Equation (3) [21], and the results show that their values were 1.51, 2.12, 1.85, and 1.33 m^2^/g, respectively, which were well consistent with values reported in the literature [22].

(3) $$S_{g}=\frac{6}{\rho D}$$

where *S_g_* is the specific surface area; *ρ* is the density of metallic W; *D* is the average particle size of W powder.

**Figure 4:**
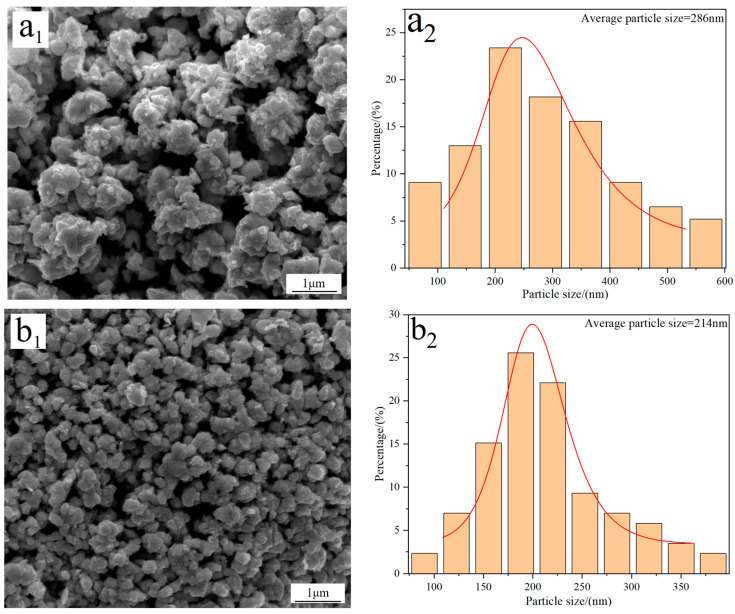

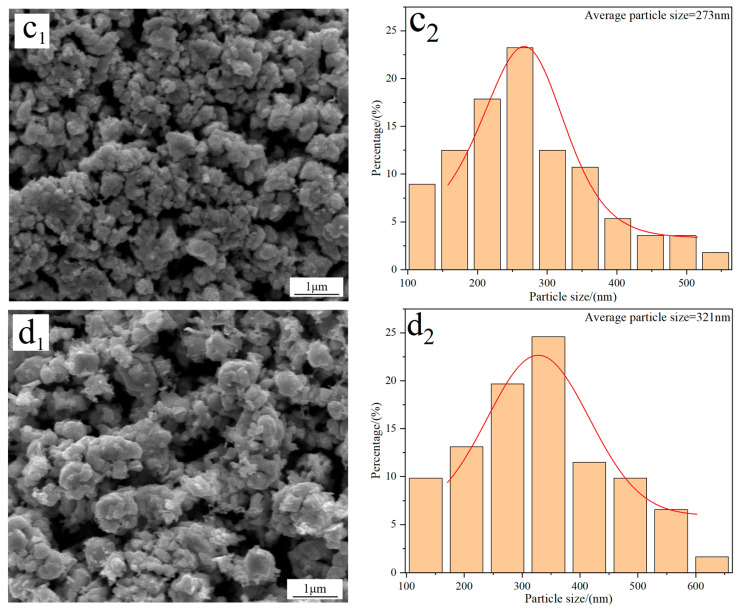
FE-SEM morphology and average particle size of W powders prepared by the H_2_ reduction of carbon-containing WO_3_ under different C/WO_3_ molar ratios: (**a_1_**,**a_2_**) 0.5; (**b_1_**,**b_2_**) 1.5; (**c_1_**,**c_2_**) 2.0; (**d_1_**,**d_2_**) 2.5. (Reduction temperature: 770 °C).

Figure 4 displays the FE-SEM morphology and average particle size of W powders prepared under different C/WO_3_ molar ratios. When the C/WO_3_ molar ratio is 0.5, the W powder exhibits a rough surface with an average particle size of 286 nm (Figure 4(a_1_,a_2_)). Increasing the C/WO_3_ molar ratio to 1.5, the morphology of W powder becomes smoother and more uniform, and a significant reduction in its particle size is observed, as seen in Figure 4(b_1_,b_2_). However, when the C/WO_3_ molar ratios increase to 2.0 and 2.5, the agglomeration phenomena of W powder become more serious, and their particle sizes increase to 273 and 321 nm, as observed in Figure 4(c_2_,d_2_). The above results indicate that the average particle size of W powder shows an initial decrease and then increase with an increase in the C/WO_3_ molar ratio. Similarly, due to the serious agglomeration phenomena of the prepared W powder, their average particle sizes measured by the FE-SEM morphology are much larger than their crystalline sizes calculated by the XRD pattern.

In addition, the specific surface areas of the above W powders prepared at the C/WO_3_ molar ratios of 0.5, 1.5, 2, and 2.5, were calculated by Equation (3), with their values of 1.08, 1.34, 1.13, and 0.96 m^2^/g, respectively. Similarly, the calculation results were also consistent with those reported in the literature [22].

## 3. Discussion

**Figure 5:**
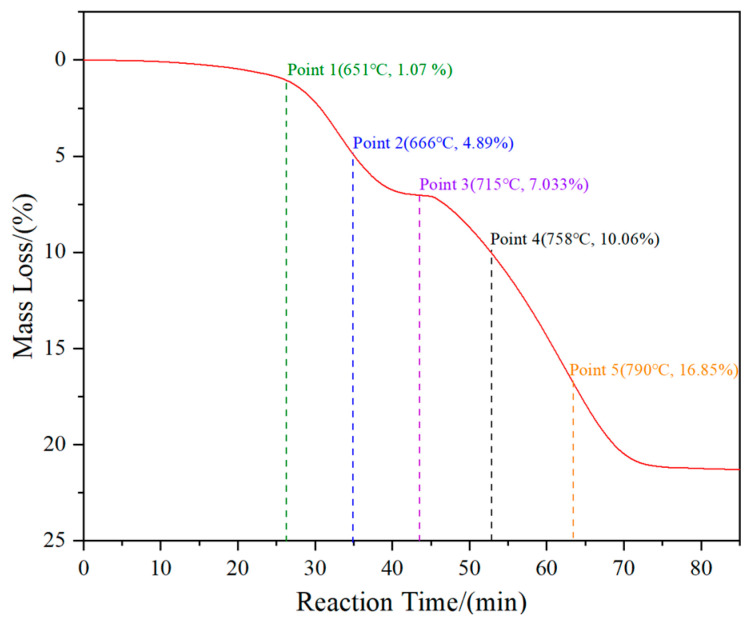
Kinetics curve of the H_2_ reduction of carbon-containing WO_3_ and the five selected intermediate products. (C/WO_3_ molar ratio: 1.0).

**Figure 6:**
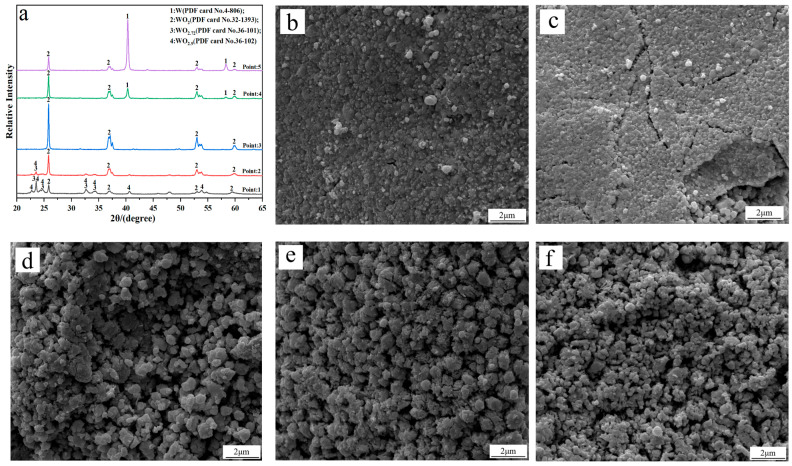
(**a**) XRD patterns of the different intermediate products; FE-SEM morphologies of the intermediate products obtained at different points marked in Figure 5: (**b**) Point 1; (**c**) Point 2; (**d**) Point 3; (**e**) Point 4; (**f**) Point 5. (C/WO_3_ molar ratio: 1.0).

To investigate the phase transition and morphology evolution regularities during the H_2_ reduction process, another series of experiments are conducted, in which different intermediate products under the C/WO_3_ molar ratio of 1.0, are obtained, as marked in Figure 5. The XRD patterns of the different intermediate products and their FE-SEM morphologies are illustrated in Figure 6. It shows that the reaction product obtained at “Point 1” are composed of WO_2.9_, WO_2.72_, and WO_2_, and its morphology is similar to WO_3_ raw material (Figure 6b). When the temperature increases to 666 °C (Point 2 marked in Figure 5), the reaction products also consist of WO_2.9_, WO_2.72_, and WO_2_, while the peak intensities of WO_2.9_ and WO_2.72_ are significantly decreased, suggesting that their relative contents are greatly reduced. In the meantime, due to the release of product gas, many cracks appear on the particle surface, as seen in Figure 6c. When the kinetics curve reaches the first plateau (Point 3 marked in Figure 5), the mass loss of sample is 7.033%, which is due to the theoretical mass loss of Reaction (4); in this situation, only the diffraction peaks of WO_2_ are detected. Both of the above results confirm that the reaction product in Point 3 is single-phase WO_2_. The morphology of product then begins to become spherical/oval shape with a smaller particle size. Increasing the temperature to 758 °C (Point 4 marked in Figure 5), the diffraction peaks of metallic W emerge, and this indicates that the partial reduction of WO_2_ to W occurs. When the temperature further increases to 790 °C, the content of WO_2_ decreases, while that of metallic W increases. Upon the completion of the reduction process, single-phase metallic W is obtained. Based on the above results, the work confirms that the total reaction pathways for the H_2_ reduction of carbon-containing WO_3_ follows WO_3_→WO_2.9_→WO_2.72_→WO_2_→W.

WO_3_ + H_2_ = WO_2_ + H_2_O (4)

Numerous papers about the reduction of W compounds under different conditions were conducted, and some of them are summarized in Table 1. From the table it can be found that when H_2_ was adopted as the reducing agent, the reaction pathways of reducing WO_3_ follows WO_3_→WO_2.9_→WO_2.72_→WO_2_→W, but when a certain number of additives were added, the pathways may have changed. Herein, the lower reduction temperature (700 °C) used in reference [14] may be due to the different experimental purposes and reaction conditions, such as the sample thickness and its physicochemical properties. From this table, it can also be observed that when CO was used as the reducing agent, the reaction pathways follows WO_3_→WO_2.72_→WO_2_→W, without the formation of WO_2.9_; however, when C_2_H_5_OH was used as the reducing agent, the reaction pathway was simplified as WO_3_→WO_2_→W. In this situation, the absence of WO_2.9_ and WO_2.72_ may be due to the high reactivity of H_2_ and CO generated via the high-temperature pyrolysis of C_2_H_5_OH. Based on the above results, this study concludes that both the reducing agents and additives had significant influences on the reduction pathways during the preparation of W powder.

**Figure 7:**
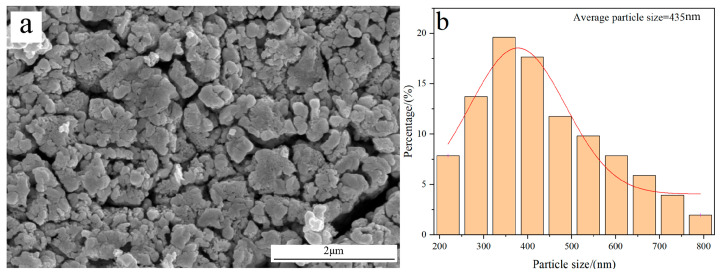
FE-SEM morphology and average particle size of W powder obtained by the H_2_ reduction of pure WO_3_ at 770 °C: (**a**) Morphology; (**b**) Particle size distribution.

Figure 7 shows the morphology and average particle size of W powder obtained by the H_2_ reduction of pure WO_3_ at 770 °C. The result reveals that the prepared W powder exhibits a rough surface structure; also, its average particle size is measured to be 435 nm, which is much larger than the value obtained by reducing carbon-containing WO_3_. The reason for the different results originates from their distinct reduction mechanisms.

During the H_2_ reduction process, the generation of water vapor is inevitable. After that, the newly formed water vapor would react with W oxides to form the chemical vapor transport (CVT) phase, which will be further reduced by H_2_ with the formation of numerous W nuclei. Then, the newly formed W nucleus can gradually grow up via continuing vapor deposition and particle collision under the high-temperature condition. That is, the H_2_ reduction of pure WO_3_ obeys the CVT mechanism, and thus the prepared W powder usually has a relatively large particle size [29].

**Figure 8:**
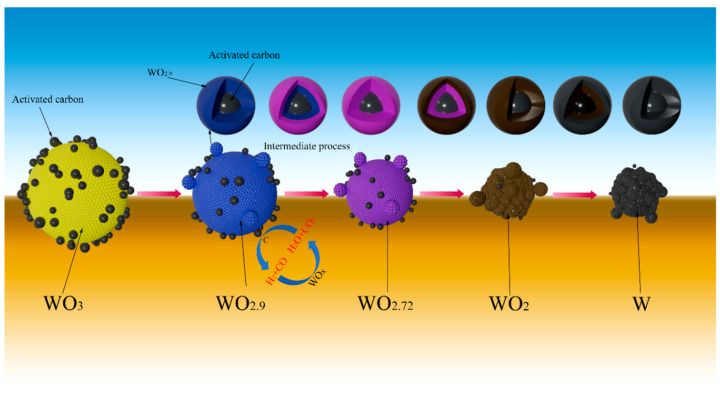
Proposed reduction mechanism of H_2_ reduction of carbon-containing WO_3_.

When a certain amount of activated carbon was added into WO_3_ raw material, the particle size of prepared W powder became much smaller. On one hand, since activated carbon was evenly mixed with WO_3_ through continuous grinding, the added activated carbon can act as nucleus sites for the formation of the subsequent metallic W, but also, due to the intense adsorption effect of activated carbon, the newly generated water vapor may adsorb on its particle surface, thus the role of CVT mechanism is greatly weakened. On the other hand, since the reaction temperature between activated carbon and water vapor is about 674 °C (obtained by the thermodynamic calculation result) [30], which is much lower than the experimental temperatures, both CO and H_2_ would form, as seen in Reaction (5). That is to say, the actual reducing gases in the reaction system have a higher reducibility than the introduced gas, thus the reduction reaction can be greatly accelerated. Moreover, the occurrence of Reaction (5) can consume lots of water vapor and weaken the role of the CVT mechanism. The above analysis suggests that adding a certain amount of activated carbon into WO_3_ raw material has a huge impact on the refinement of the prepared W powder, and the possible reduction mechanism of carbon-containing WO_3_ with H_2_ can be simply illustrated by Figure 8.

C + H_2_O = CO + H_2_  Δ*G^θ^* = −143.39 *T* + 135,811 (5)

## 4. Materials and Experimental Procedures

### 4.1. Materials

**Figure 9:**
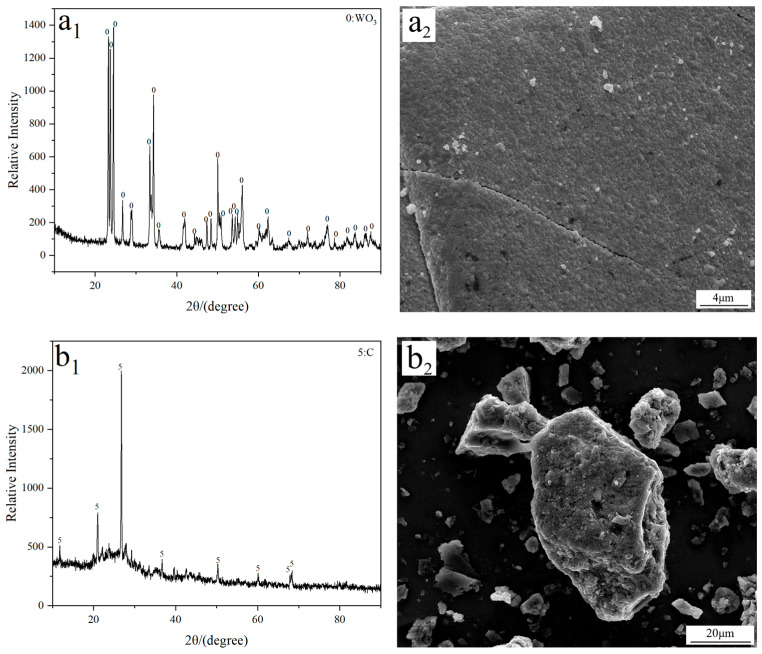
(**a_1_**) XRD and (**a_2_**) FE-SEM results of the used WO_3_ raw material; (**b_1_**) XRD and (**b_2_**) FE-SEM results of the used activated carbon.

Analytical grade tungsten trioxide (WO_3_; 99.9%; Tianjin Obokai Chemical Reagent Co., Ltd., Tianjin, China) and activated carbon (Tianjin Fuchen Chemical Reagent Co., Ltd., Tianjin, China) were used as the W source and additive, respectively. FE-SEM imaging reveals that both WO_3_ and activated carbon exhibit a block morphology with a large particle size, as observed in Figure 9. To investigate the influence of C/WO_3_ molar ratio on the reaction behavior, the mixture of activated carbon and WO_3_ with different C/WO_3_ molar ratios (0.5, 1.0, 1.5, 2.0, and 2.5) were prepared.

### 4.2. Experimental Procedures

**Figure 10:**
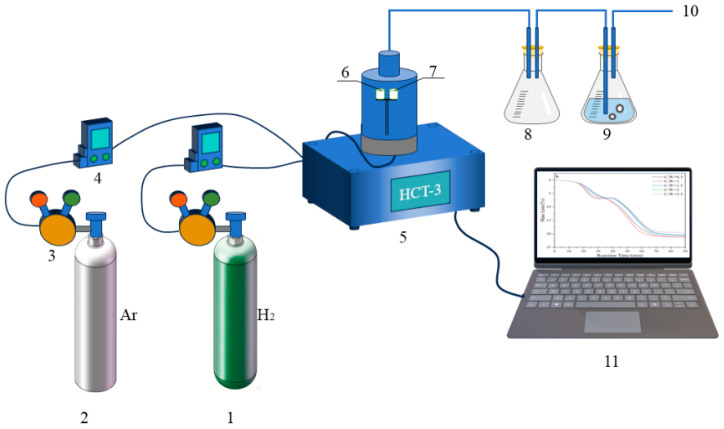
Schematic diagram of the experimental apparatus: 1, H_2_; 2, Ar; 3, gas pressure reducer; 4, gas flow meter; 5, HCT-3 thermal analysis system; 6, calibrated alumina crucible; 7, experimental alumina crucible; 8, empty beaker flask; 9, beaker flask with water; 10, exhaust gases; 11, data collection device.

In order to investigate the reaction behaviors of H_2_ reduction of carbon-containing WO_3_, the HTC-3 thermal analysis system equipped with a thermos-gravimetry (TG) microbalance (Beijing Hengjiu Experimental Equipment Co., Ltd., Beijing, China) that boasts a precision of ±0.1 μg, was used. The schematic diagram of the experimental apparatus is illustrated in Figure 10. In each of the experimental runs, 100 mg of sample mixture was first placed into the alumina crucible “7” (Φ7 mm × 7 mm). A “dead-burnt” identical alumina crucible “6” was used as the standard reference material. After the sample-containing crucible “7” was positioned on the supported holder and the balance was in a stable state, Ar gas with the flow rate of 100 mL/min was first introduced to remove the air out. After that, H_2_ gas with the same flow rate was introduced. In the meantime, the furnace was heated to different temperatures (740, 770, 800, and 830 °C) under the heating rate of 10 °C/min and was maintained at that temperature for 4 h. Upon completion of the reaction, the furnace was turned off and the sample cooled to room temperature. Finally, we collected the reaction products into a sealable bag for characterization.

### 4.3. Sample Characterization

X-ray diffraction analyzer (XRD; D8 Advance, AXS Corporation, Bruker, Germany; operation voltage: 30 kV; operation current: 20 mA; scanning rate: 10°/min) was used to identify the phase composition of sample, and field emission scanning electron microscope (FE-SEM; Nova 400 NanoSEM, FEI Corporation, Hillsboro, OR, USA; Operation voltage: 15 kV) was used to observe its morphological structure.

## 5. Conclusions

(1) The phase transition law of the H_2_ reduction of carbon-containing WO_3_ was given as WO_3_→WO_2.9_→WO_2.72_→WO_2_→W.
(2) Due to the co-actions of adsorbents and nucleating agents, as well as the weakness of CVT mechanism, the addition of a certain amount of activated carbon into WO_3_ raw material could contribute to the refinement of the W powder.
(3) The optimal conditions for preparing ultrafine W powder by the H_2_ reduction of carbon-containing WO_3_ was given as reduction temperature of 770 °C and C/WO_3_ molar ratio of 1.0; under the conditions, ultrafine W powder with an average particle size of approximately 146 nm was prepared.

## Figures and Tables

**Table 1:** List of the reduction processes of W compounds under different conditions.

Reference	W Sources	Temperature	Reducing Agent	Particle Size	Reaction Pathways
Wang et al. [14]	WO_3_ (99.95%)	700 °C	H_2_	/	WO_3_→WO_2.9_→WO_2.72_→WO_2_→W
Estupinan-Donoso et al. [15]	WO_3_ (99.9%)	750~972 °C	H_2_ (5%)	0.486–0.983 μm	WO_3_→WO_2.9_→WO_2.72_→WO_2_→W
Choi et al. [23]	(NH_4_)_6_W_12_O_39_*x*H_2_O(99.99%) Y(NO_3_)_3_⋅6H_2_O(99.8%)	800 °C	H_2_	0.403 μm	WO_3_→WO_2.9_→WO_2_→W
Lv et al. [24]	WO_3_ (99.9%) Y(NO_3_)_3_(99%)	765~927 °C	CO	/	WO_3_→WO_2.72_→WO_2_→WC
Lv et al. [25]	(NH_4_)6H_2_W_12_O_40_·5H_2_O (99.0%) Y(NO_3_)_3_·6H_2_O(99.9%)	800~900 °C	H_2_	/	WO_3_→WO_2.72_→WO_2_→W
Salleh et al. [26]	WO_3_ (99.9%) Ni(NO_3_)_2·_6H_2_O(99%)	800 °C	40%CO-10%H_2_	/	WO_3_→WO_2.72_→WO_2_→W
Li et al. [27]	WO_3_ (99.9%)	937 °C	15%CO-85%CO_2_	/	WO_3_→WO_2.9_→WO_2.72_→WO_2_→W
Cetinkaya et al. [28]	WO_3_	927~1277 °C	C_2_H_5_OH	1.6–3.3 μm	WO_3_→WO_2_→W
This work	WO_3_ Activated carbon	740~830 °C	H_2_	0.146–0.321 μm	WO_3_→WO_2.9_→WO_2.72_→WO_2_→W

## Data Availability

The raw data can be provided upon reasonable request.
